# Supplementary material for: Prognostic Implications of the Residual Tumor Microenvironment after Neoadjuvant Chemotherapy in Triple-Negative Breast Cancer Patients without Pathological Complete Response
Source: Cancers (Basel). 2023 Jan 18;15(3):597. doi: 10.3390/cancers15030597 (PMC9913578; doi:10.3390/cancers15030597)

Supplementary Table S1. Antibodies and probes used in the immunohistochemical (IHC) and *in situ* hybridization (ISH) analyses (clone, source and dilution).

**Antibody name (dilution)***	**Dilution clone**	**Supplier (code)**
CD4 (RU)	4B12	Dako, Santa Clara, CA, USA (IR649)
CD8 (RU)	C8/144B	Dako, Santa Clara, CA, USA (IR623)
FOXP3	236A/E7	CNIO, Madrid, Spain
CD57 (1:20)	NK1	Thermo Fisher Scientific, Waltham, MA, USA (Zymed-18-0167Z)
CD68 (RU)	KP1	Dako, Santa Clara, CA, USA (IR609)
CD1a (RU)	010	Dako, Santa Clara, CA, USA (IR069)
CD21 (RU)	1F8	Dako, Santa Clara, CA, USA (IR608)
CD83 (1:20)	1H4b	LEICA-Novocastra (NCL-CD83)
CD34 class II (RU)	QBEnd/10	Dako, Santa Clara, CA, USA (FLEX IR632)
CD31 (RU)	JC70A	Dako, Santa Clara, CA, USA (FLEX IR610)
CD15 (1:50)	CARB-3	Dako, Santa Clara, CA, USA (IR 062)
HLA-Dr (1 :100)	TAL.1B5	Dako, Santa Clara, CA, USA (M-0746)
CD138 (RU)	MI15	Dako, Santa Clara, CA, USA (IR642)
**Target molecules**	**Probe name**	**Supplier (code)**
CXCL13	NM_006419	Affymetrix, Inc., Santa Clara, CA (VA1-15914)
IL6 (interferon, beta 2)	NM_000600	Affymetrix, Inc., Santa Clara, CA (VA1-13526)
IL10	NM_000572	Affymetrix, Inc., Santa Clara, CA (VA1-10840)
IL15	NM_000585	Affymetrix, Inc., Santa Clara, CA (VA1-12546)
MMP1	NM_002421	Affymetrix, Inc., Santa Clara, CA (VA1-10114)
MMP9	NM_004994	Affymetrix, Inc., Santa Clara, CA (VA1-16679)
MMP12	NM_002426	Affymetrix, Inc., Santa Clara, CA (VA1-17343)
MUC1	NM_002456	Affymetrix, Inc., Santa Clara, CA (VA1-14606)
TNF-α	NM_000594	Affymetrix, Inc., Santa Clara, CA (VA1-10481)
NOTE. * all mouse monoclonal, RU: ready-to-use		

Figure S1. Representative tissue microarray (TMA) stained tumor sections of the 22 tumor microenvironment (TME) markers studied. Cell markers were analyzed by immunohistochemistry (IHC): CD4, CD8, FOXP3, CD57, CD68, CD15, CD21, CD1a, CD83, HLA-DR, CD31, CD34 and CD138; genetic markers were analyzed by chromogenic *in situ* hybridization (ISH): CXCL13, IL-6, IL-10, IL-15, MMP1, MMP-9, MMP12, MUC1 and TNF-α.

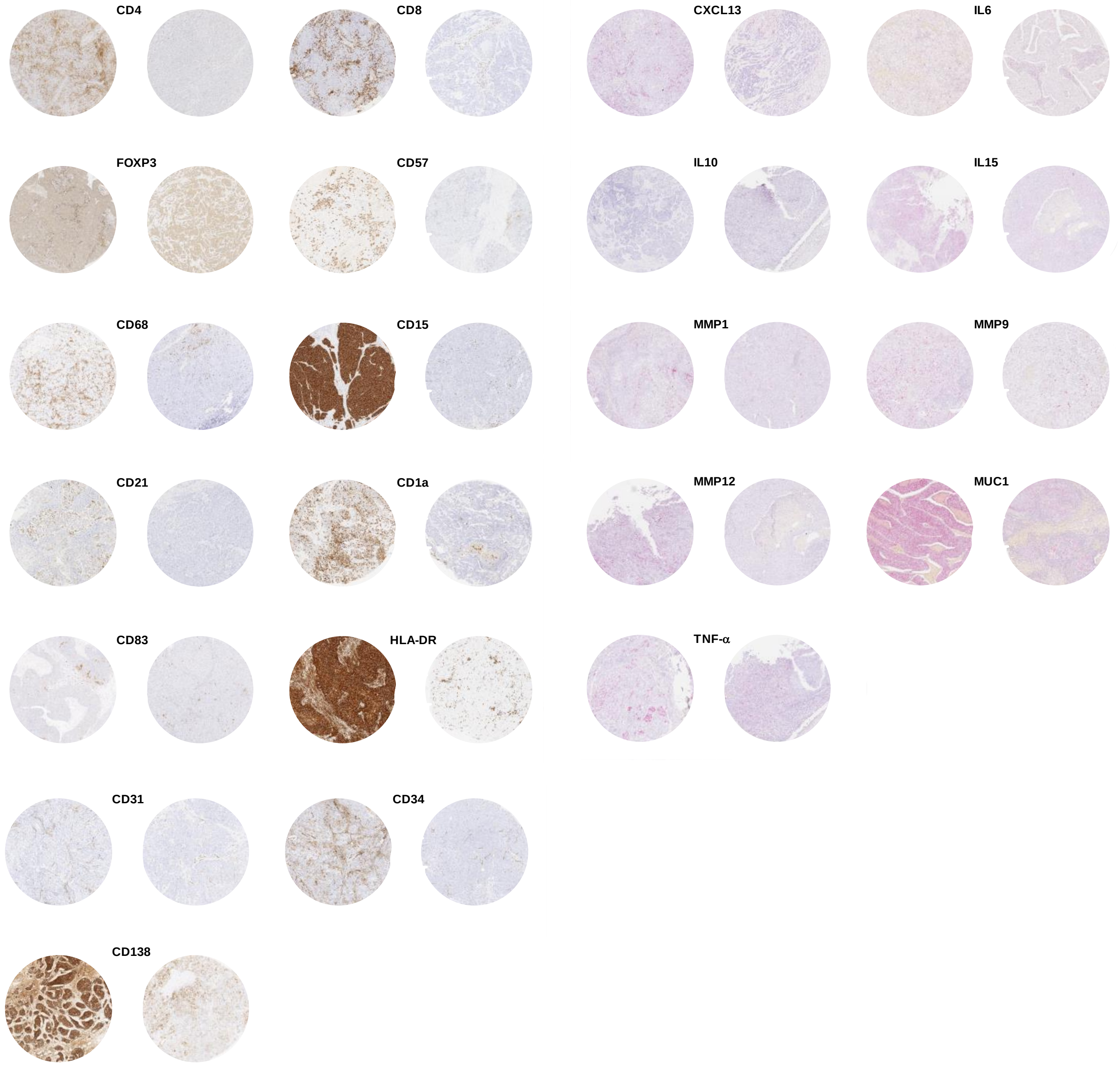

Figure S2. Kaplan–Meier analysis of five-year overall survival (OS) among all patients.

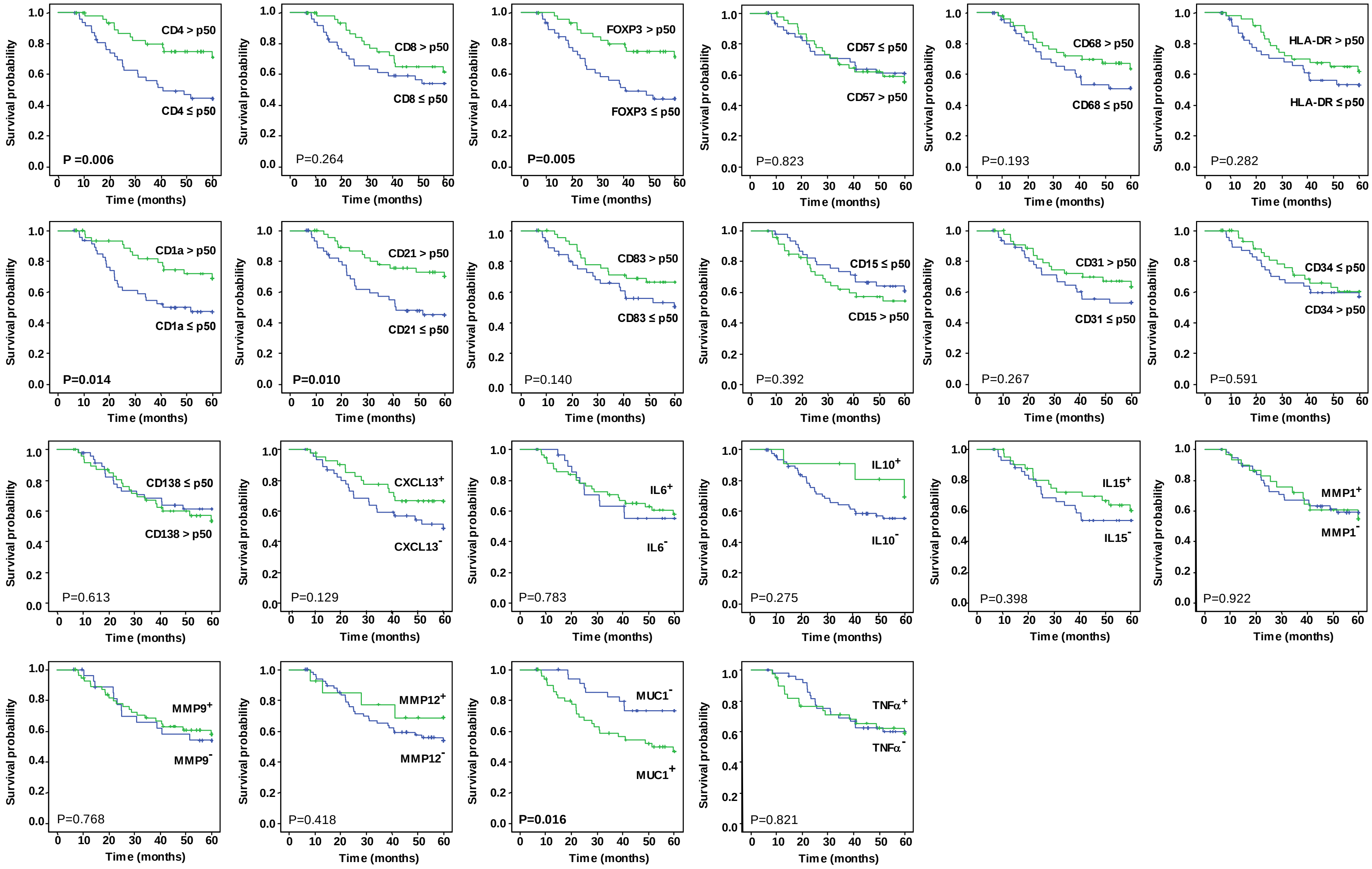

Figure S3. Kaplan–Meier analysis of five-year relapse-free survival (RFS) among all patients.

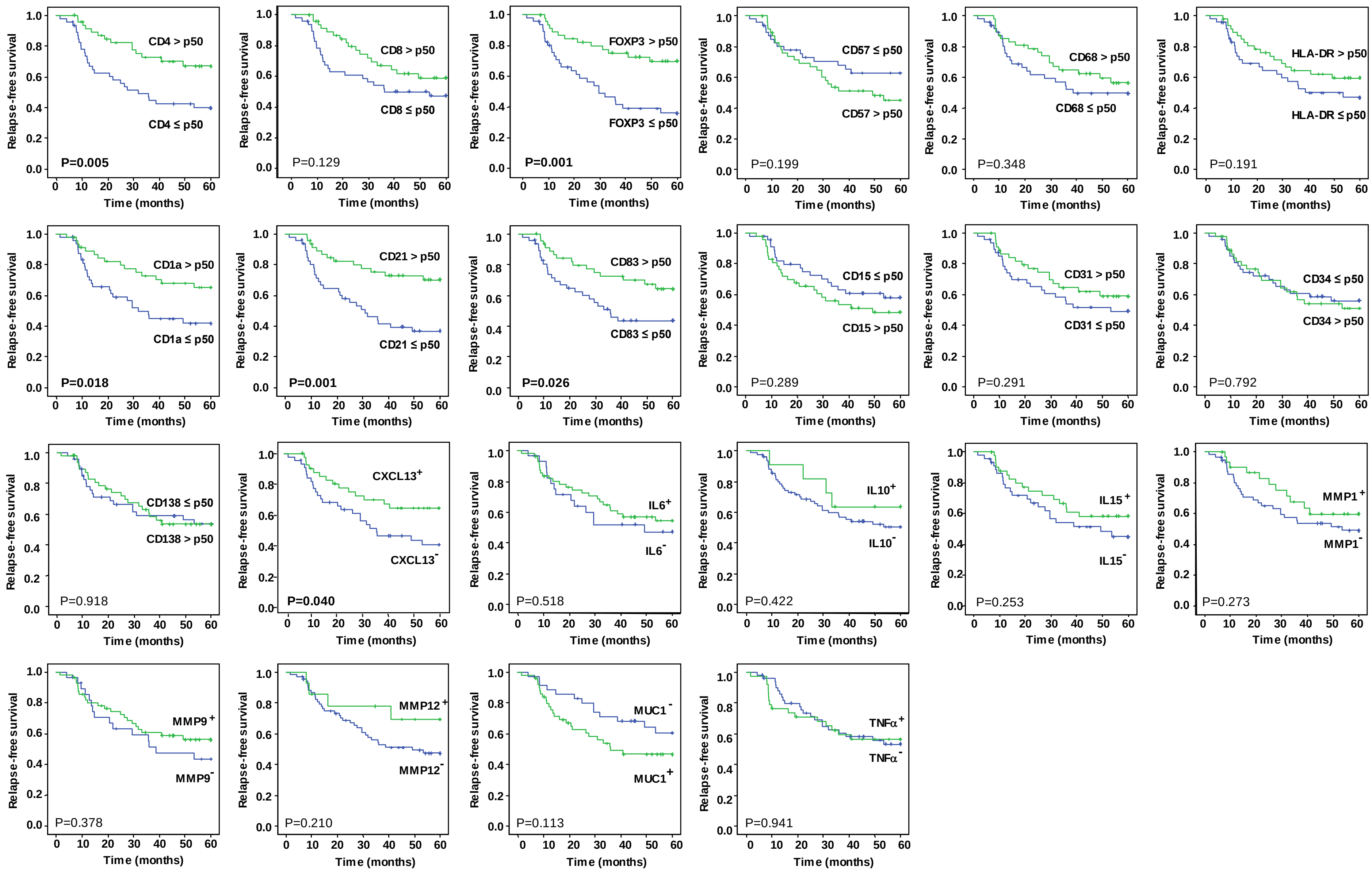

Figure S4. Superimposed Kaplan–Meier curves of five-year overall survival (OS) by tumor microenvironment (TME) markers by nodal status at diagnosis. The continuous lines show the OS of patients with immunohistochemistry (IHC) immune marker concentrations higher than the median, or positive mRNA expression levels for markers determined by *in situ* hybridization (ISH). The dashed lines show the OS when IHC immune marker concentrations were lower than the median, or when mRNA was not expressed for ISH markers. Significance levels for the log-rank test are indicated in the figures.

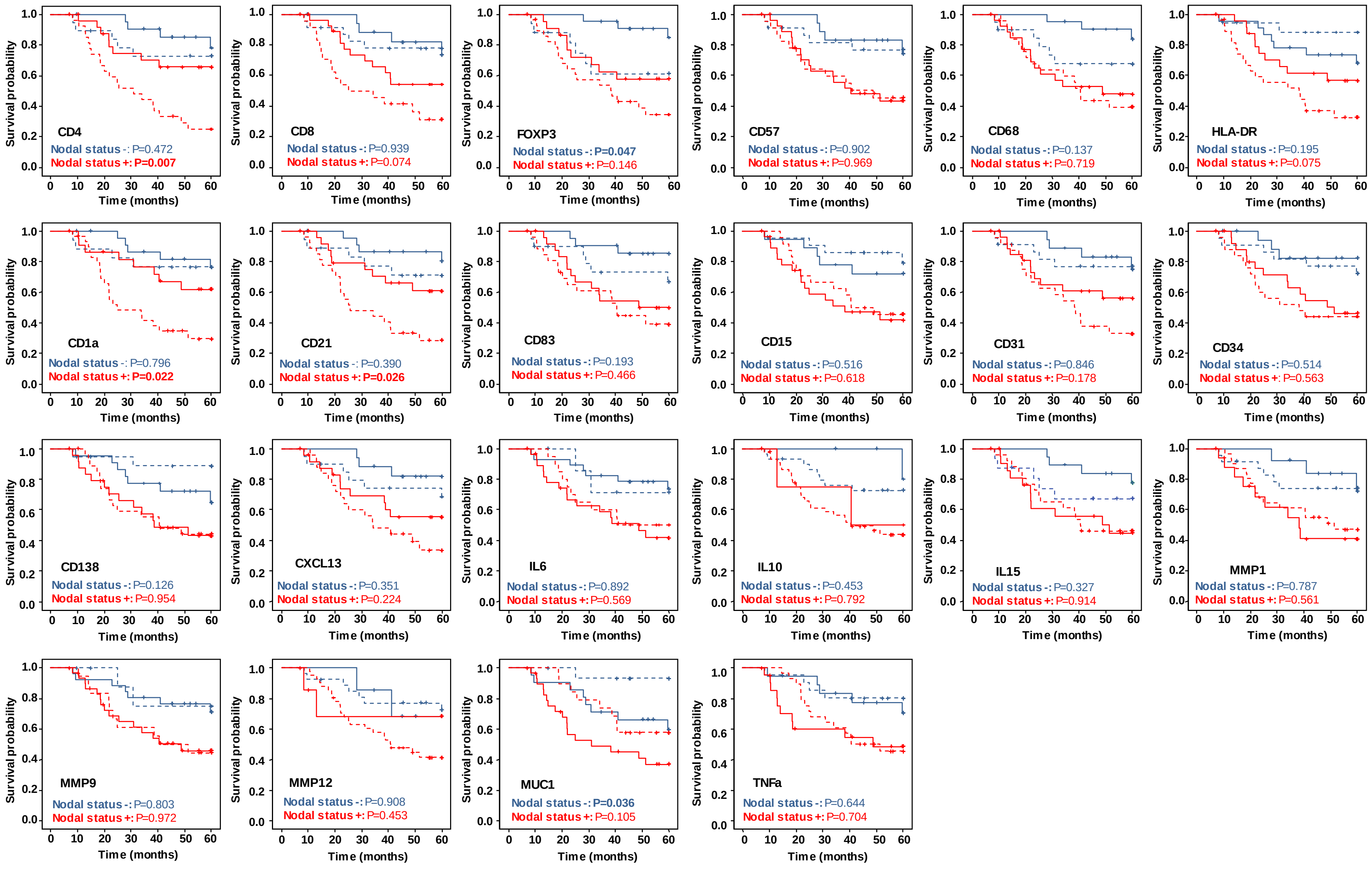

Figure S5. Superimposed Kaplan–Meier curves of five-year relapse-free survival (RFS) by tumor microenvironment (TME) markers by nodal status at diagnosis. The continuous lines show the RFS of patients with immunohistochemistry (IHC) immune marker concentrations higher than the median, or with positive mRNA expression levels for markers determined by *in situ* hybridization (ISH). The dashed lines show the RFS when IHC immune marker concentrations were lower than the median or when mRNA was not expressed for ISH markers. Significance levels for the log-rank test are indicated in the figures.

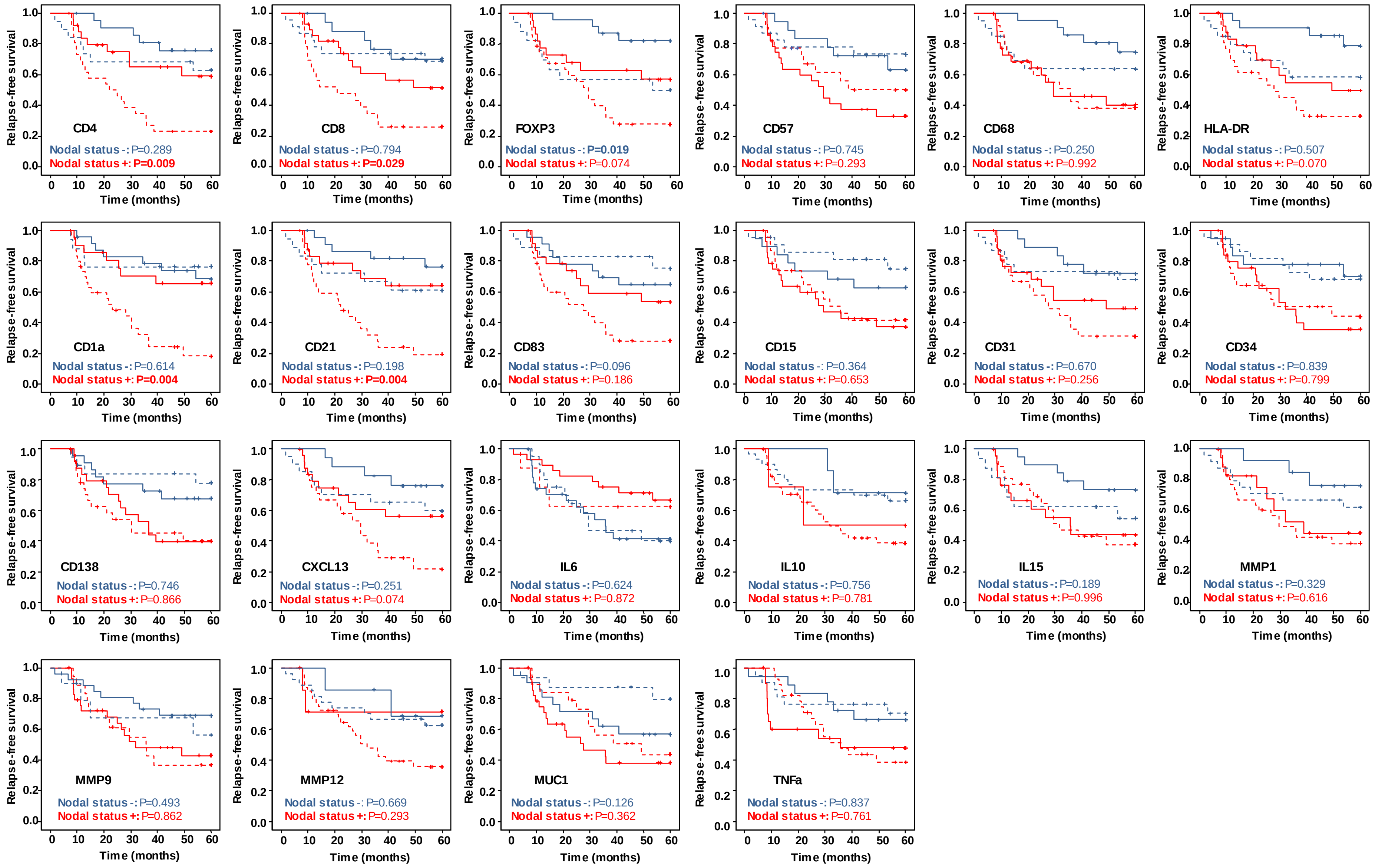

Figure S6. Superimposed Kaplan–Meier curves of five-year overall survival (OS) by tumor microenvironment (TME) markers by type of response. The continuous lines show the OS of patients with immunohistochemistry (IHC) immune markers concentrations higher than the median, or with positive mRNA expression levels for markers determined by *in situ* hybridization (ISH). The dashed lines show the OS when IHC immune markers concentrations were lower than the median or when mRNA was not expressed for ISH markers. Significance levels for the log-rank test are indicated in the figures.

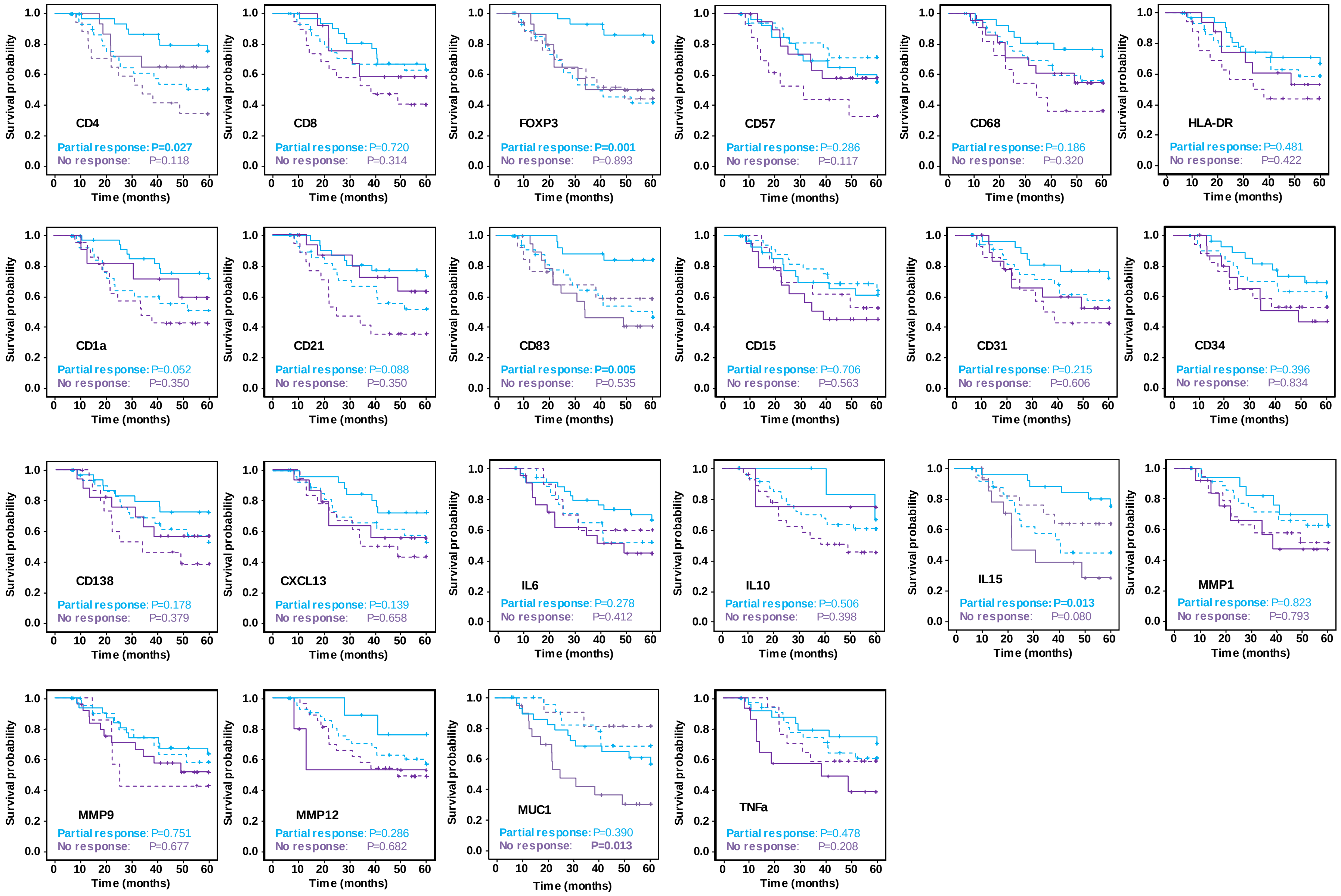

Figure S7. Superimposed Kaplan–Meier curves of five-year relapse-free survival (RFS) by tumor microenvironment (TME) markers by type of response. The continuous lines show the RFS of patients with immunohistochemistry (IHC) immune markers concentrations higher than the median, or with positive mRNA expression levels for markers determined by *in situ* hybridization (ISH). The dashed lines show the RFS when IHC immune markers concentrations were lower than the median or when mRNA was not expressed for ISH markers. Significance levels for the log-rank test are indicated in the figures.

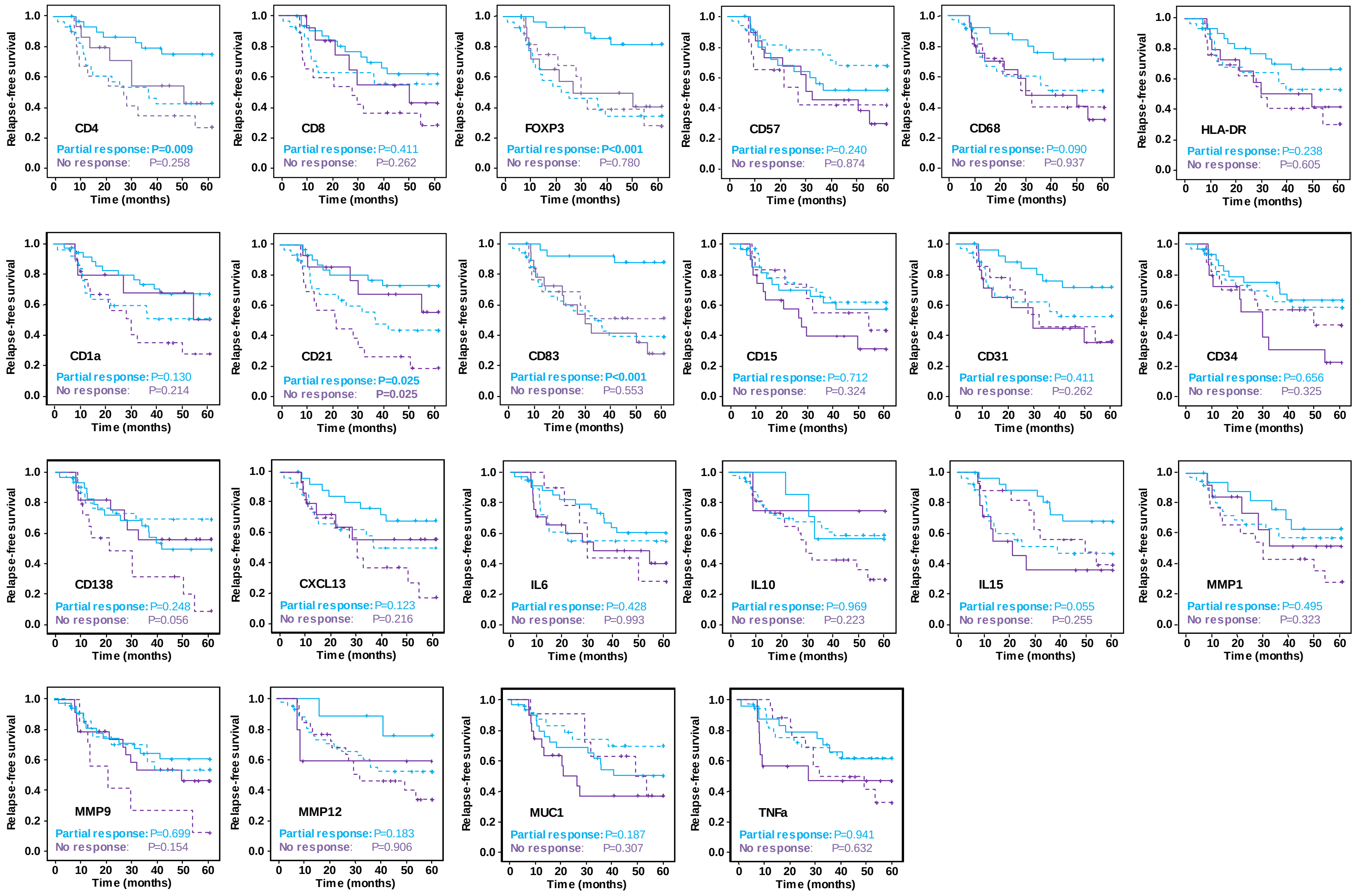

Supplement: Supplementary file 1 [file cancers-15-00597-s001.zip › cancers-2111110-supplementary.pdf]
